# Quantification of EGFR-HER2 Heterodimers in HER2-Overexpressing Breast Cancer Cells Using Liquid-Phase Electron Microscopy

**DOI:** 10.3390/cells10113244

**Published:** 2021-11-19

**Authors:** Diana B. Peckys, Daniel Gaa, Niels de Jonge

**Affiliations:** 1 Clinic of Operative Dentistry, Periodontology and Preventive Dentistry, University Hospital, Saarland University, 66421 Homburg, Germany; Diana.Peckys@uks.eu; 2 INM—Leibniz Institute for New Materials, 66123 Saarbrücken, Germany; s8dagaaa@stud.uni-saarland.de; 3 Department of Physics, Saarland University, 66123 Saarbrücken, Germany

**Keywords:** cancer cell heterogeneity, breast cancer, gastric cancer, EGFR, HER2, EGFR/HER2 heterodimers, correlative light- and liquid-phase electron microscopy, single molecule detection, absolute quantification

## Abstract

Currently, breast cancer patients are classified uniquely according to the expression level of hormone receptors, and human epidermal growth factor receptor 2 (HER2). This coarse classification is insufficient to capture the phenotypic complexity and heterogeneity of the disease. A methodology was developed for absolute quantification of receptor surface density *ρ*_R_, and molecular interaction (dimerization), as well as the associated heterogeneities, of HER2 and its family member, the epidermal growth factor receptor (EGFR) in the plasma membrane of HER2 overexpressing breast cancer cells. Quantitative, correlative light microscopy (LM) and liquid-phase electron microscopy (LPEM) were combined with quantum dot (QD) labeling. Single-molecule position data of receptors were obtained from scanning transmission electron microscopy (STEM) images of intact cancer cells. Over 280,000 receptor positions were detected and statistically analyzed. An important finding was the subcellular heterogeneity in heterodimer shares with respect to plasma membrane regions with different dynamic properties. Deriving quantitative information about EGFR and HER2 *ρ*_R_, as well as their dimer percentages, and the heterogeneities thereof, in single cancer cells, is potentially relevant for early identification of patients with HER2 overexpressing tumors comprising an enhanced share of EGFR dimers, likely increasing the risk for drug resistance, and thus requiring additional targeted therapeutic strategies.

## 1. Introduction

EGFR and HER2 are overexpressed membrane proteins and therapeutic targets in many different types of cancer, for instance, in breast and gastric cancers [1,2]. These receptors can form homodimers, as well as heterodimers, resulting in the activation of their intracellular tyrosine kinase and the subsequent initiation of downstream signaling cascades, promoting cell proliferation, survival, and dissemination [3,4]. In cancer cells, the expression and interaction of EGFR and HER2 belong to the phenotypic heterogeneity of cancer cells [5,6,7,8], which leads, in combination with genetic heterogeneity, to drug resistance and disease progression [9,10,11]. The interest in analyzing phenotypic heterogeneity in patient tumors as a predictive biomarker is rising [7,12,13]. Besides receptor overexpression, cancer cells also exhibit a striking deregulation between the spatial distribution, interaction, and signaling strength of membrane receptors, including EGFR [14] and HER2 [15,16,17,18], which correlates with an increased risk of resistance against targeted therapeutics [19]. We, therefore, aimed to develop a method for the absolute quantification of EGFR and HER2 *ρ*_R_, the HER2/EGFR *ρ*_R_ ratio, the corresponding dimerization profile, and the associated heterogeneity thereof, including the specific, subcellular localization in distinct plasma membrane regions.

**Figure 1:**
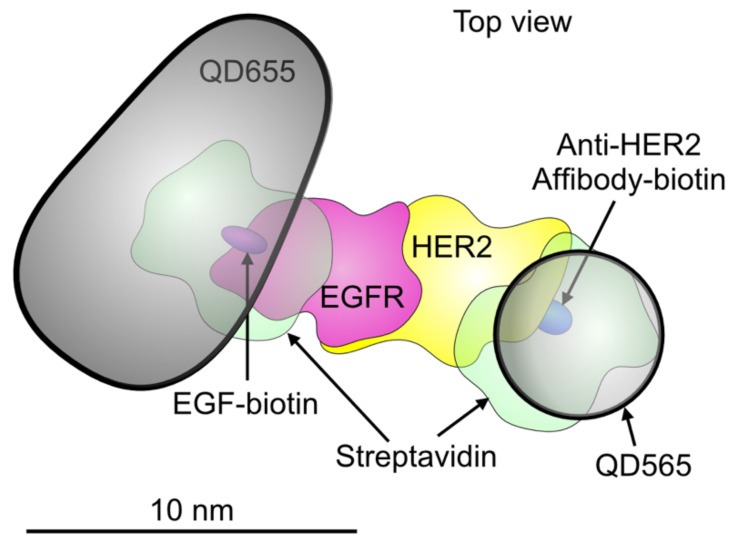
Schematic model depicting an EGFR-HER2 heterodimer with two bound quantum dots (QDs). A QD655 (the bullet-shaped core is shown) is bound to EGFR via streptavidin and an EGF-biotin linker. A smaller QD565 (spherical shape) is bound to HER2 through a streptavidin and an Affibody-biotin compound. The published molecular model of the EGFR-HER2 heterodimer was used [45]. The other used molecular structures (5WB7, 3MZW, and 1STP) were obtained from the Protein Data Base. Dimensions are drawn to scale.

Application of LM-based methods for absolute quantification of *ρ*_R_ values of EGFR or HER2 in overexpressing cancer cells [20,21], is prone to diverse artifacts and biases [22,23], often due to steric hindrance of the labels since the *ρ*_R_ may reach 10^3^/μm^2^ [24,25,26,27,28]. Immuno-gold labeling, traditionally used in most transmission electron microscopy-based studies, is generally associated with an extended distance between the gold nanoparticle and its protein target [29,30], impairing the localization precision as needed to detect the various dimeric forms. In addition, usage of immuno-gold labeling for absolute quantification is excluded due to steric hindrance, intrinsic gold-nanoparticle clustering [31], and the use of polyclonal antibodies easily leading to label:protein ratios larger than one [30]. To be able to quantify receptor *ρ*_R_, as well as dimer percentages, our labeling approach used binding proteins with smaller mass, by an order of magnitude, than antibodies, namely EGF and an anti-HER2 affibody, in combination with two different types of quantum dots, QD655 and QD565 (see Figure 1). A two-step labeling protocol prevented label-induced receptor clustering. Prior to labeling, the cells were chemically fixed with formaldehyde and glutaraldehyde, to prevent any ligand-induced dimerization or clustering that can occur during labeling of unfixed samples or with samples fixed using only formaldehyde, a detailed discussion of this topic can be found in our earlier publication [32]. We performed correlative LM and LPEM [33], using STEM, of intact, chemically fixed SKBR3 cells. This cell line is an established model for HER2 overexpressing breast cancer [34], and has on average 4-fold lower EGFR-, and ~50-fold higher HER2 expression levels than normal breast tissue [35]. Automated particle detection and analysis of the STEM images yielded single-molecule position data of >9,300 labeled EGFR, and >275,000 labeled HER2, from 41 cells. With the knowledge of the determined labeling efficiency *η* [20], the absolute values of local receptor *ρ*_R_ were calculated from the QD’s *ρ*. To measure the receptor fractions in monomers and dimers, the data from the STEM images were analyzed with the pair correlation functions, *g*(*r*), for homodimers, and *g_2_*(*r*)*,* for heterodimers, and the results were compared with simulated images of dimers at known percentages. The data shown here demonstrate that the method works for the analysis of homodimers and heterodimer fractions at a sub-cellular level. However, it should be noted that general conclusions about the distribution and heterogeneity of these oligomers in breast cancer cells should be supported by data using other cells lines and ideally also cells from patient biopsy samples.

## 2. Materials and Methods

### 2.1. Materials

Biotin conjugated anti-HER2 Affibody, from Affibody AB, Bromma, Sweden. Dulbecco’s phosphate-buffered saline (DPBS), from Lonza, Cologne, Germany. QD565- and QD655 streptavidin conjugates, non-essential amino acids (NEEAs) 100× solution, fetal bovine serum (FBS), Dulbecco’s Modified Eagle’s Medium (DMEM) GlutaMAX with high glucose and pyruvate, endogenous biotin blocking kit, EGF biotin-, and EGF Fluorescein conjugates, from Thermo Fisher Scientific GmbH, Dreieich, Germany. H_2_O, ethanol, acetone, all high-pressure liquid chromatography (HPLC) grade, phosphate-buffered saline (PBS) 1× solution pH 7.4, biotin free and molecular biology grade bovine serum albumin fraction V (BSA), glycine, and sodium cacodylate trihydrate (CB), from Carl Roth GmbH + Co. KG, Karlsruhe, Germany. 0.01% aqueous Poly-L-lysine solution, biotin, boric acid, sodium tetraborate, sucrose, electron microscopy grade 25% glutaraldehyde solution, superfibronectin, from Sigma-Aldrich, Munich, Germany. Electron microscopy grade formaldehyde 16% solution, from Science Services GmbH, Munich, Germany. Quantum™ FITC-5 MESF (Polystyrene beads FITC conjugated calibration beads), from Polyscience Europe GmbH, Hirschberg an der Bergstraße, Germany. Normal goat serum (GS), from Rockland Immunochemicals, Gilbertsville, PA, USA. 35 mm 4-well compartment glass-bottom dishes and well plates (6, 24, 48, and 96) for tissue culture, from Greiner Bio-One GmbH, Frickenhausen, Germany. Silicon microchips with a 50 nm thick electron transparent silicon nitride (SiN) window with dimensions of 120 × 700 µm^2^, from Norcarda, Edmonton, AB, Canada. Multi-layer graphene (3–5 layers, polymethyl methacrylate trivial transfer graphene), from ACS Materials, Pasadena, CA, USA. CellStripper, from Corning, New York, NY, USA.

### 2.2. Mammalian Cell Culture

The SKBR3 cell line (HTB-30), serving as a model for HER2 overexpressing breast cancer, was purchased from ATCC, Wesel, Germany. Cells were cultured in growth medium (DMEM-GlutaMAX with 10% FBS and 1% NEAAs) and kept in a CO_2_ incubator, in a 5% CO_2_ water-saturated, air atmosphere, at 37 °C. Cells were passaged after reaching 60–85 confluency, approximately twice a week, and used up to passage number 25. Cells were authenticated by single nucleotide polymorphism analysis (Mulitplexion, Friedrichshafen, Germany) and tested for contamination by mycoplasma.

### 2.3. Coating of Microchips and Cell Culture Microscope Dishes

Microchips were prepared for cell seeding as described earlier [36]. In brief, microchips were incubated in HPLC-grade acetone, ethanol, H_2_O, rinsed in ethanol, air-dried and plasma cleaned with argon and oxygen, to get hydrophilic surfaces. The plasma cleaning was also applied to dishes. For enhancing cellular adherence, the surfaces of the microchips and the bottom of dishes were submerged with poly-L-lysine, rinsed twice with water, incubated with superfibronectin, diluted 5 µg/mL in PBS, for two hours at 37 °C. After two rinses with PBS, the microchips were placed in a 96-well plate, filled with serum-free DMEM, similarly, the compartments of dishes were filled with serum-free DMEM.

### 2.4. Cell Seeding on Microchips or Cell Culture Microscope Dishes

A detailed description was published earlier [36], briefly, SKBR3 cells were non-enzymatically detached with cell stripper, re-suspended in growth medium, counted, and diluted to approximately 1 × 10^5^ cells/mL. 100 µL of cell suspension, containing approximately 1 × 10^4^ cells, was added to each microchip in a 96-well plate. 1 mL of cell suspension, containing approximately 1 × 10^5^ cells, was added to each compartment in dishes. After 5 min, the cell density on the surface was checked and, if needed, more cell suspension was added. The samples were placed in the CO_2_ incubator for 2 h. Afterwards, microchips were transferred to new wells pre-filled with growth medium, and the medium in dishes was replaced with fresh growth medium. Cells were allowed to grow for 48 h before applying serum-starvation (for ~18 h) to enhance the expression level of EGFR [37] and to reduce cell-to-cell variation in protein abundance caused by the different cell cycle phases [38].

### 2.5. Dual Labeling of EGFR and HER2

The procedures applied for fixation and growth factor labeling of the cells were performed as described earlier [32]. In brief, the applied two-step fixation started rinsing the cells 1× with serum-free medium, and 1× with CB (0.1 M cacodylate buffer, 0.1 M sucrose, pH 7.4), followed by a 5 min incubation with 3% formaldehyde (in CB), and another 5 min in 3% formaldehyde and 0.2% glutaraldehyde (in CB.) After rinsing 1× with CB, 3× with PBS, a 10 min quenching step with 1 M glycine (in PBS), and 1× rinsing with PBS, the growth factor receptors EGFR and HER2 were labeled using specifically binding small peptides and two different types of QD.

First, the EGFR receptors were labeled by a 10-min incubation with EGF-biotin (400 nM (in PBS/BSA1%). After two rinses and a 5 min incubation with PBS/BSA1%, the cells were incubated with streptavidin-conjugated quantum dots 655 (QD655) (20 nM in PBS/BSA1%) for 12 min, followed by two rinses, and a 5 min incubation with PBS/BSA1%. Accessible streptavidin-binding sites on the cells were then saturated by a 5 min incubation with streptavidin solution (from the endogenous biotin blocking kit). After 3× rinsing with PBS, excess free biotin-binding sites were saturated by a 5 min incubation with biotin solution (30 µg/mL in PBS/BSA1%) followed by 3× rinsing with PBS.

HER2 was labeled next, by first blocking unspecific affibody binding sites with 1 M glycine/GS1% (in PBS/BSA1%) for 10 min, before performing a 10 min incubation with anti-HER2 Affibody-biotin (200 nM in PBS/BSA1%/GS1%). The cells were then rinsed 2× and incubated for another 5 min with PBS/BSA1%. Afterwards, the cells were incubated for 12 min with the smaller, streptavidin-conjugated quantum dots 565 (QD565) (20 nM in PBS/BSA1%), followed by two rinses and a 5 min incubation with PBS/BSA1%.

Immediately afterwards, the cells were imaged with LM, and the cells on microchips were rinsed with CB, fixed with 2% GA (in CB) for 10 min, rinsed 1× with CB, 3× with PBS, and stored in PBS/BSA1% at 4 °C until LPEM was performed.

### 2.6. Determination of the Labeling Efficiency for EGFR

For determining the labeling efficiency of EGFR labeled with biotinylated EGF and QD655, a similar approach as previously described for the determination of the QD-labeling efficiency of membrane-bound HER2 in the SKBR3 cell population [20] was applied. The methodology based on the same principles for protein quantification as used for quantitative flow cytometry [39]. To adapt the procedure for EGFR, the labeling protocol assuring ~100% labeling efficiency was performed with FITC-conjugated EGF. More detailed information can be found in the Supplementary Information, including Additional Materials and Methods: *Determination of the labeling efficiency* *ρ_R_* for membrane-bound HER proteins, Figures S2 and S3.

### 2.7. Differential Interference Contrast and Fluorescence Microscopy

LM was performed with an inverted microscope (DMi6000B, Leica, Wetzlar, Germany). Cells on microchips were imaged with the cells touching the glass bottom (microchips upside down) in a dish filled with 1 mL PBS/BSA1%. Images with 1392 × 1040 pixels were acquired with a 20× (HC PL FLUOTAR L 20×/0.40 DRY) objective. The channels used were: differential interference contrast (DIC), fluorescence for QD565 (ex. 425/50 nm; dichroic 505 nm; em. 565/30 nm), fluorescence for QD655 (ex. 425/50 nm; dichroic 510 nm; em. 655/30 nm) and fluorescence for FITC (filter L5 ex. 460–500 nm/em. 512–542 nm). After LM, cells on microchips were prepared for electron microscopy with a 10-min fixation in 2% glutaraldehyde, followed by rinsing 1× with CB, and 3× with PBS. Microchip samples were then stored in PBS/BSA1% containing NaN_3_ 0.02%, at 4 °C until electron microscopy was performed.

### 2.8. Graphene Enclosure of Microchips for Electron Microscopy

Immediately before electron microscopy, the microchip samples were covered by multilayer graphene. Details on the preparation and transfer of graphene sheets can be found elsewhere [36]. In brief, the graphene sheet was released from its salt support and floated in a beaker filled with water. A metal loop was used to capture the floating graphene in a small water droplet. The microchip to be examined with electron microscopy was rinsed 3× with water and positioned directly under the loop, cells facing the graphene. Under visual control through a binocular microscope, the excess water between the graphene sheet and the cells on the microchip was carefully removed with filter paper.

### 2.9. Scanning Transmission Electron Microscopy

A graphene-covered microchip was placed in a standard electron microscope holder and imaged with STEM at 200 keV beam energy (ARM 200F, JEOL, Akishima, Japan). Pixel images (2048 × 2048) were recorded for overview purposes at *M* = 800 × (pixel size of 0.13 µm), and label analysis at *M* = 120,000 × (pixel size of 0.83 nm) using image acquisition software (Digital Micrograph, Version 3.30.2016.0, Gatan, CA, USA). The condenser lens aperture was 20 µm, the probe size (2C) corresponded to a probe current of 175 pA, the beam convergence angle was 13.2 mrad, the STEM detector opening angle *β*_in_ − *β*_out_ = 68–280 mrad and the pixel dwell time was 18 µs. The maximal electron dose was calculated to be 280 e^−^Å^−2^, which lies under the limit of radiation damage for these samples. A detailed demonstration of how correlative microscopy was used to identify and image the same membrane regions with LM and LPEM, can be found in our earlier publication [32].

### 2.10. Particle Analysis

Images recorded at *M* = 120,000× were analyzed with locally developed image processing scripts (Fiji ImageJ, version 1.52p, NIH, USA) [17]. To detect the individual particles in a STEM image, a pipeline of several image processing steps was performed, defined in a locally developed ImageJ Plugin. First, a bandpass filter was used to filter out noise and low-frequency background variation. The image was then binarized using the Maximum Entropy option provided by ImageJ. The particles were then detected using the Analyze Particles tool in ImageJ. The expected areas of large and small particles were calculated based on their given diameters, leading to a lower and upper threshold for each of the particle types. The Analyze Particle tool was then used twice with the respective thresholds for the small and large particles in order to detect and distinguish them. The minimal and maximal diameters were 10 ± 2 nm, respectively. 5 ± 2 nm for the larger QDs (QD655) and smaller ones (QD566), respectively. Output files contained the x/y coordinates for each detected QD in separate lists for QD565 and QD655. These results were also used to calculate the QD densities of each image.

The lateral (x, y) positions were statistically analyzed with local design software to calculate the pair correlation function g(*r*) for label pair center-to-center distance *r* [40]:

(1) $$g\left(r\right)=\frac{1}{\pi r\rho^{2}\gamma \left(r\right)}\sum_{i=1}^{n}\sum_{j=i+1}^{n}k\left(r-\left|x_{i}-y_{j}\right|\right),$$

where *n* is the number of particles o, *ρ* is the surface density and $x\in {\mathbb{R}}^{n_{1}\;x\;2}$ and $y\in {\mathbb{R}}^{n_{2}x\;2}$ matrices containing the 2D-coordinates of each particle, such that ***x****_i_* gives the coordinates of the i^th^ particle and ***y****_j_* the coordinates of the j^th^ particle. | | is the L2 (Euclidean) norm.

The spatial correlation between particles of two types in heterodimers was calculated using the bivariate pair correlation function [41]:

(2) $$g_{2}\left(r\right)=\frac{1}{2\pi r\rho_{1}\rho_{2}\gamma \left(r\right)}\sum_{i=1}^{n_{1}}\sum_{j=1}^{n_{2}}k\left(r-\left|x_{i}-y_{j}\right|\right),$$

where *n*_1_ and *n_2_* are the number of particles of type 1 and type 2, with respective *ρ* values *ρ*_1_ and *ρ*_2_. **x***_j_* gives the coordinates of the i^th^ particle of type 1 and **y***_j_* the coordinates of the j^th^ particle of type 2.

As kernel function *k*(*x*), the function proposed by Fiksel et al. was used [42]:

(3) $$k\left(x\right)=\left\{\begin{array}{c} \frac{3}{4\varepsilon }\left(1-\frac{x^{2}}{\varepsilon }\right),\;\left|x\right|\leq \varepsilon \\ 0,\;else \end{array}\right.$$

where *ε* is called bandwidth. The covariance function defined as [43]:

(4) $$\gamma \left(r\right)=hw-\frac{2\left(h+w\right)}{\pi }r+\frac{r^{2}}{\pi },$$

where *h* and *w* are the height and the width of the image, respectively, were used to compensate for image edges.

Distances smaller than 7.5 nm were excluded from the calculation in order to avoid counting overlapping nanoparticles. The calculation assumed the labels to be positioned approximately planar. The output of the analysis was a histogram of *g*(*r*) or *g*_2_(*r*) with a bin width in *r* of 10 nm. The bandwidth in the calculation was set equal to the bin width. The data of different images were averaged for the same regional type of plasma membrane, i.e., LMPs, clustered regions, and lamellipodia. The average was weighted by the *ρ*_R_ and, as a consequence, cells with a lower number of labels were less strongly weighted in the average than cells with more labels.

### 2.11. Statistical Analysis

Statistical significance was tested with the one-way analysis of variance (ANOVA), followed by pots hoc analysis with the Games–Howell Test, a nonparametric approach to compare combinations of groups with unequal variances and sample sizes, using α = 0.05. Differences were considered significant at *p* < 0.05 and are indicated in the Figures as follows: * *p* < 0.05, ** *p* < 0.01 and *** *p* < 0.001.

### 2.12. Random Simulation

Random simulations were done using a locally designed plugin for ImageJ, creating images with random simulated particle distributions of round particles with the dimensions of QD565 and QD655 (as published in [44]) and with the mean label pair distance, as found by g(*r*) analysis. The plugin enabled the selectable input of parameters such as the *ρ*_R_ of EGFR and HER2, for which the determined values of the real STEM images were used, the calculated values for *η* and two estimated parameters, a peak width parameter *σ*, and the share of particles in dimers. In the process, the large particles were placed first, each of them at a random position. In the second step, a portion of the small particles was placed in the vicinity of some of the large particles, creating heterodimers. The position of each second particle was determined by a randomly generated angle and distance to the first particle. The angle was generated from a homogenous distribution. The distance was obtained from a Gaussian distribution, using a specified mean = 10 nm for QD565, respect. 15 nm for QD655. The value for the peak width *σ*, reflecting the experimentally observed variation of the label distances in dimers, was set to 5, except for fits of EGFR homodimers in FSM and LMP, where values of 9 yielded better results and a value of 1 for HER2 homodimers in lamellipodia. In the last step, the rest of the small particles were placed, again at random positions, representing the small monomers. When placing a new particle, it was made sure that none of them were overlapping (if they were, the new particle was not placed at that position, but a new random position was generated instead). Hereby, the software distinguished the hard shell and the soft shell of the particles. Within the hard shell (equals the particle diameter), no overlap was possible. Within the soft shell, it was decided randomly whether the particle position was accepted or not, where the probability of acceptance increased with the distance of the particles. The thickness of the soft shell was chosen to be 2.5 nm, as derived from earlier measurements of STEM images of negatively stained QDs [44]. The soft shell was modeled using the following transition function:

(5) $$t\left(s\right)=1-e^{-{\left(slope*s\right)}^{2}}$$

where $t\in \left[0,\left.1\right)\right.$ was the probability of rejecting the particle. The *slope* was a fixed parameter given by $slope=\frac{1}{softshellthickness}$. The parameter $s\in \left[0,\;2softshellthickness\right]$ set the amount of overlap between the two particles.

## 3. Results

### 3.1. Determination of the ρ_R_ of EGFR and HER2 in Functionally Distinct Subregions of the Plasma Membrane

The two different QDs, namely QD655 and QD565, were used for the labeling of EGFR and HER2, respectively, a scheme depicting a dual labeled receptor dimer is shown in Figure 1. The used QD differ in size, shape, and fluorescence emission so that the receptors are distinguishable in both LM and EM.

**Figure 2:**
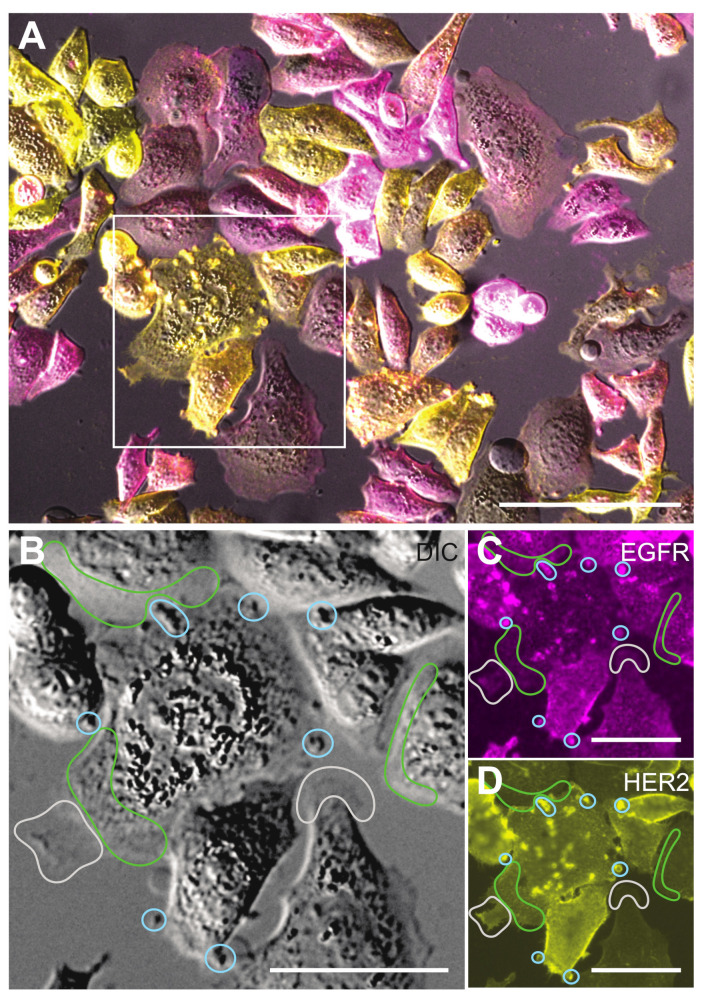
Light microscopy (LM) of SKBR3 cells dual-labeled for EGFR- and HER2. (**A**) LM overlay image from the differential interference contrast (DIC)-, and the fluorescence signals of the EGFR-QD655 label (magenta), and the HER2-QD565 label (yellow). The QD655 channel was adjusted in a more sensitive manner than the QD565 channel to compensate for the lower *ρ*_R_ of EGFR compared to HER2. The cells in the marked square display an increased concentration of both receptors in delimited areas (bright spots) of the plasma membrane. (**B**) DIC-image detail of the boxed area in (**A**), with marked examples of three functionally different regions of the plasma membrane, being large plasma membrane protrusions (LMPs), enclosed by blue lines, lamellipodia, which are flat and slowly oscillating protrusions at the rim of a cell, marked by grey lines, and the areas without a special structure in the LM images, classified as fine-structured membrane (FSM) areas, were delimited by green lines. (**C**) Same region as in (**B**), but then for the fluorescence signal from QD655-labeled EGFR, which is particularly bright in LMP. (**D**) Same region as in (**B**), fluorescence signal of QD565-labeled HER2. The similarity of the bright spots in (**C**,**D**) indicates a correlated distribution of both receptors in LMPs. Scale bars: 100 µm in (**A**), 50 µm in (**B**–**D**).

To analyze the interaction between EGFR and HER2, five functionally different protein conformations were detected in an experiment, (i) EGFR monomer, (ii) EGFR homodimer, (iii) HER2 monomer, (iv) HER2 homodimer, and (v) EGFR-HER2 heterodimer (Figure 1). Fluorescence images were recorded to map the spatial distributions of both proteins in the cells and served for later navigation and orientation during LPEM. The typical phenotypic heterogeneity of cancer cells [10,46] is shown in Figure 2, displaying a representative group of dual-labeled SKBR3 cells. The cell phenotypes differed in size, shape, HER2- and EGFR *ρ*_R_, and in the ratios of HER2 and EGFR*ρ*_R_. In many cells, both receptors accumulated in distinct regions of the plasma membrane, for instance in the cell marked with a square in Figure 2A, where areas with accumulated receptors appeared as bright spots in the fluorescence images. Inspection of parallel recorded DIC images revealed that these areas were protrusions of the plasma membrane, which belong to the class of dynamic membrane ruffles [47]. Different sizes of membrane ruffles exist ranging from 0.5–5 µm [17,47]. We have shown earlier that membrane ruffles are the preferred locations of HER2 homodimers [47,48]. Here, we analyzed membrane protrusions of lateral dimensions of 2–5 μm as these were clearly visible in the DIC images. They were termed large membrane protrusions (LMP). A different type of plasma membrane region is a lamellipodium, a submicron-thin, several μm wide membrane region at the cell border, with even surface topography, often oscillating between a protruding and a retracting motion. The remaining regions of the plasma membrane showed homogeneous, low to intermediate fluorescence intensities, and irregular, fine-structured surface features, mostly <1 μm, in the DIC channel. These regions were categorized as a third membrane region type and termed fine-structured membrane (FSM).

**Figure 3:**
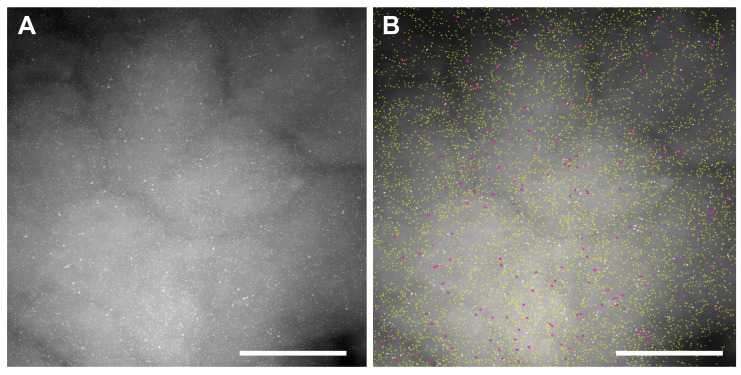
Exemplary STEM image from a large membrane protrusion (LMP). (**A**) Original scanning transmission electron microscopy (STEM) image 120,000×. Individual quantum dots (QDs) are visible as bright spots, created by larger QD655 bound to EGFR, and many smaller QD565 bound to HER2. (**B**) Same image as in (**A**), but after automated image processing detecting the labels, outlining QD655 (EGFR) in cyan and QD565 (HER2) in yellow, and saving the x,y position information of all detected labels. Scale bars 500 nm.

To measure the subcellular *ρ*_R_ of both receptors, STEM images were recorded of selected areas, each depicting ~3 μm^2^ of plasma membrane from 41 cells at 120,000× magnification, (Table 1). Figure 3A shows an exemplary STEM image from an LMP region, displaying many bright spots, indicating the locations of QD565 bound to HER2, and fewer, larger QD655 bound to EGFR, both labels were bound with a max. 1:1 ratio to their targets. Automated image processing and analysis detected and discriminated QD655 from QD565 (Figure 3B), determined their lateral (x,y) positions, and calculated both *ρ*_R_. The underlying *ρ*_R_ of the respective receptors was obtained by correcting the *ρ*_R_ of the QD for the labeling efficiency fraction of *η*. For QD565 labeling of HER2, *η* = 0.8 [20] (Figures S2 and S3), and for EGFR labeled with QD655, *η* = 0.4 (Figure S3).

**Figure 4:**
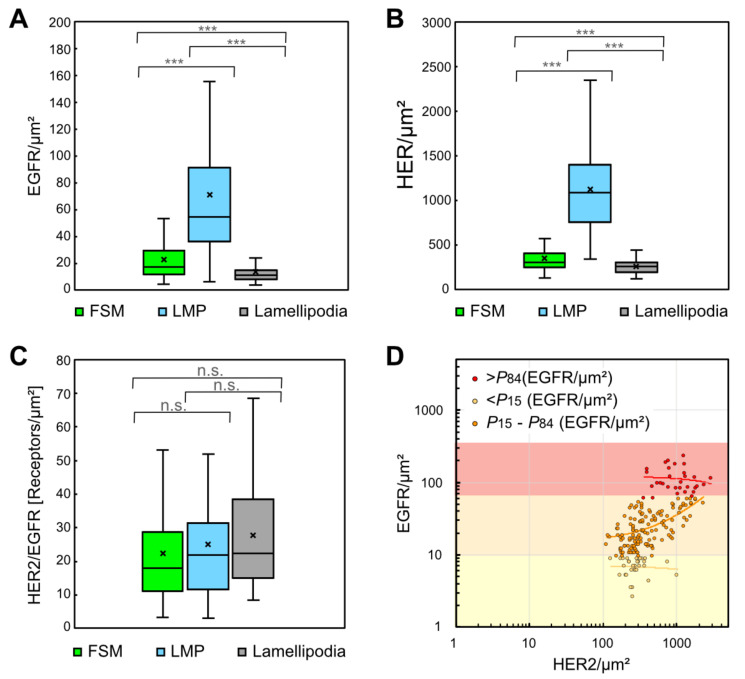
Distributions of local EGFR and HER2 *ρ*_R_, and HER2/EGFR ratios in three different plasma membrane regions. (**A**) Box plots of EGFR *ρ*_R_ determined from data of STEM images from FSM regions (number of analyzed images *n* = 102), LMP (*n* = 64), and lamellipodia (*n* = 31). Differences between the regions were statistically significant. (**B**) Box plots for HER2 *ρ*_R_ for the same regions as in (**A**), revealing similar statistic differences between the membrane regions as found for EGFR. (**C**) Box plot of the HER2/EGFR *ρ*_R_ ratios in the three plasma membrane regions. The ratios were not statistically different between the three regions. (**D**) Dot plot of corresponding EGFR and HER2 *ρ*_R_ in all acquired STEM images analyzed to test a correlation between EGFR and HER2 *ρ*_R_. The data set was divided into three groups, depending on the EGFR *ρ*_R_. Linear regression (lines) of the subgroup representing an intermediate level of EGFR *ρ*_R_ (*P*15–*P*84, orange dots) yielded a Pearson correlation coefficient R^2^ = 0.4, thus indicating a weak correlation. However, the data of the upper 15% (>*P*84, red dots) and the lower 15% (<*P*15, yellow dots) of EGFR *ρ*_R_ yielded R^2^ < 0.02, respectively *R*^2^ < 0.01, thus lacked any correlation. *p* values: *** < 0.001.

As shown in Figure 4A for EGFR, and in Figure 4B for HER2, the LMP areas exhibited several fold higher mean values for *ρ*_R_ of both receptors than those of FSM and lamellipodia. However, the three membrane regions showed similar HER2/EGFR *ρ*_R_ ratios with a mean of approx. 25 (Figure 4C), which is close to the ratio of 20 reported for pooled averages of serum-starved and antibody-labeled SKBR3 cells [49]. To derive a quantitative measure of heterogeneity for EGFR and HER2 *ρ*_R_, the coefficient of variation (*CV*) was calculated for each type of membrane region and for the local *ρ*_R_ ratios between both receptors. The *CV* for both receptor’s *ρ*_R_s ranged between 0.3 and 0.8, *CV* values for the *ρ*_R_ ratios were between 0.7 and 1.0 (see Table 1). Statistical analysis by ANOVA and subsequent post hoc analysis showed significant differences between the region-specific EGFR-, respect. HER2 *ρ*_R_ (*p* < 0.001). In contrast, the *ρ*_R_ ratios of EGFR/HER2 were not significantly different (*p* = 0.3–0.8).

To examine a possible correlation between the *ρ*_R_ of both receptors, all measured EGFR *ρ*_R_s were plotted against their corresponding HER2 *ρ*_R_ (Figure 4D). When the data were split into three groups, depending on the local EGFR *ρ*_R_*,* linear regression analysis revealed a low correlation (Pearson’s correlation coefficient (*R*^2^) = 0.4) in the intermediate group with EGFR/μm^2^ values between 9 and 59, which includes the data from the 15th to the 84th percentile (*P*15–*P*84), but no correlation appeared in the upper and lower ranges of EGFR *ρ*_R_ (>*P*84 and <*P*15) (see Figure 4D). No correlation was found by linear regression analysis through the complete set of data (*R*^2^ < 0.3, see Figure S1A), nor within each of the three types of membrane regions (all *R*^2^ < 0.3, see Figure S1B). In addition, linear correlations were not found between cell size, serving as a measure for cell proliferation, and average EGFR or HER2 *ρ*_R_ per cell, however, such a correlation appeared between cell size and the total number of HER2 per cell (see Figure S1C,D).

### 3.2. Quantification of EGFR and HER2 in Heterodimeric, Homodimeric and Monomeric States

**Figure 5:**
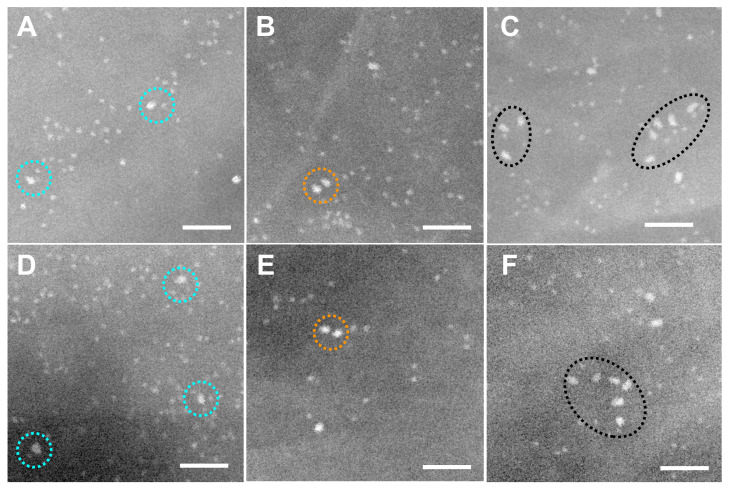
Exemplary details from STEM images of dual labeled SKBR3 cells showing EGFR and HER2 in dimers and clusters. The smaller, round, white dots are the cores of QD565 bound to HER2, while the larger, bullet-shaped, white dots are QD655 bound to EGFR. Labeled HER2 dominated and formed dimers as well as larger clusters. (**A**,**D**) Examples of dual-labeled heterodimers (marked with dotted, turquoise circles), consisting of labeled EGFR adjacent to labeled HER2 (label-to-label distances 15–20 nm). (**B**,**E**) EGFR homodimers (highlighted with orange, dotted circles) and HER2 homodimers (blue dotted circles). (**C**,**F**) EGFR was also occasionally found in clusters (black, dotted ovals). All images were recorded with LPEM at *M* = 120,000×, scale bars: 50 nm.

In order to determine if receptors were in a dimeric state, STEM images were recorded from selected membrane areas and the spatial distribution of the labels were analyzed. Figure 5 shows six representative details of STEM images recorded from dual-labeled SKBR3 cells with the smaller QD565 bound to HER2 and the larger QD655 to EGFR. Paired labels with an intra-pair center-to-center distance of 10 to 30 nm were classified as double-labeled receptor dimers [17,48]. Examples of putative EGFR heterodimers are highlighted in Figure 5A and D, EGFR pairs can be seen in Figure 5B and E and clusters comprising >2 labels of EGFR are marked in Figure 5C,F. It should be kept in mind that *η* < 1 for QD labeling, and so the number of underlying receptors is higher than the number of QDs. This also means that not all EGFR homo- and heterodimers are discernable as label pairs, or in clusters, due to dimers with only one label or with no label.

**Figure 6:**
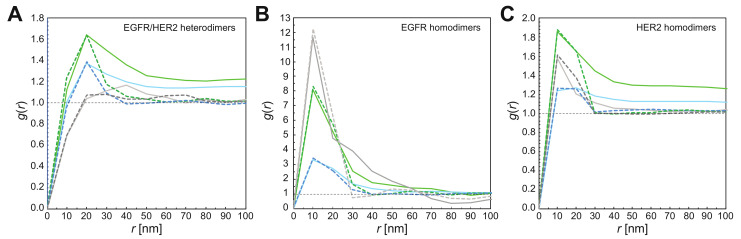
Analysis of label pairs using the computed pair correlation function *g(r)* and bivariate pair correlation function *g_2_(r)* of EGFR and HER2 experimental position data, and of label positions in simulated images. (**A**) Graphs of *g_2_(r)* for EGFR-HER2 heterodimers from pooled QD655 and QD565 position data as detected in the three different plasma membrane regions (solid lines). Graphs of *g_2_(r)* of simulated images of QD655 and QD565 are included as well (dotted lines). These were generated using the same parameters as for the experimental data but with randomly positioned EGFR-HER2 heterodimers. (**B**) *g(r)* of EGFR homodimers of experimental data, and of corresponding simulations. (**C**) *g(r)* of the HER2 data sets with corresponding simulations.

Since labels may also have been positioned at close proximity due to random chance, a statistical analysis was needed to test for the presence of receptor dimers. QD-label pairs can be identified as belonging to dimers [17,48] through statistical analysis of the label pair distance *r* via the pair correlation function *g*(*r*) [43]. This function measures the probability of finding a range of *r* values in the data. The function is normalized such that *g*(*r*) = 1 for a random particle distribution and *g*(*r*) > 1 reflecting clustering, for example, dimerization [17,50]. The *g*(*r*) of QD-labeled EGFR or HER2 had a typical peak with a maximum at *r* = 10–20 nm, depending on the size of the QD used (Figure 6), consistent with the label center-to-center distance for labeled receptor dimers (Figure 1). The peak extended up to 30 nm. Such peak would not be present for randomly distributed receptors, since pre-clustering was ruled out in previously published control experiments [17,44], the peak thus indicates the presence of homodimers. For analyzing heterodimers, we used an extended bivariate pair correlation function between pairs of different labels *g*_2_(*r*).

To elucidate the activation profile and the interaction of both receptors on a subcellular level, we determined the fractions of receptors in the dimeric or monomeric state in the three distinct membrane regions. After sorting the QD position data sets into the three categories of imaged plasma membrane regions, a *g*(*r*) analysis was performed on the pooled data of each region type. Figure 6 shows the resulting *g*(*r*) and *g*_2_(*r*) graphs for EGFR-HER2 heterodimers, EGFR-homodimers, and HER2 homodimers. All curves, except the one for EGFR-HER2 heterodimers in lamellipodia, had peaks above the random level between *r* = 10–20 nm, indicating the presence of receptor dimers.

The height of the *g*(*r*) peak depends on the percentage of labeled receptors found in dimers, and on the *ρ*_R_ of the labels, with higher densities decreasing the peak height for the same fractional share. To determine the actual percentages of receptors assembled in dimers, the measured *g*(*r*) and *g*_2_(*r*) peak values were compared with simulated random distributions of single labels and label pairs at the experimentally measured QD *ρ*_R_ and the known values of *η*. Simulated distributions were generated using estimated values for the dimer fraction, and *g*(*r*) peak width. The resulting *g*(*r*) curves of these simulated label distributions were then computed. Both estimated parameters were varied until the *g*(*r*) of the simulated distributions (dotted lines in Figure 6) matched the peak height of the respective *g*(*r*) of the experimental data (solid lines in Figure 6). Note that in the *g*(*r*) of the simulated data, the dimer peak returned to 1 for *r* > 30 nm while in the *g*(*r*) of the experimental data, the peak stayed >1 beyond that range, indicating the presence of clusters of >2 labeled receptors, as well as bends in the plane of the plasma membrane through ruffles and other protrusions [47]. Presumably, clusters > 2 originated from random positioning of single receptors, homodimers, and heterodimers, although certain regions of the plasma membrane may have exhibited higher densities of receptors, for example, in so-called lipid rafts [51]. For the quantification of homodimer and heterodimer *ρ*_R_, we assumed clustering was purely random, and the plasma membrane was assumed to be flat so that only the peak region of *g(r)* around *r* = 20 nm was considered.

**Figure 7:**
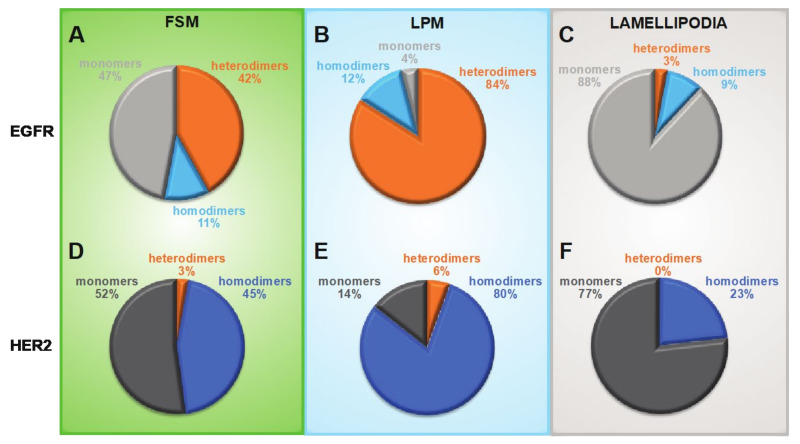
EGFR and HER2 distribution profiles of heterodimers, homodimers, and monomers in three characteristic membrane regions. (**A**–**C**) Pie charts showing the determined fractions of monomeric, hetero- and homodimeric fractions of EGFR. (**D**–**F**) Pie charts for the correspondingly determined fractions of HER2 in heterodimers, homodimers and monomers.

The percentages of heterodimeric and homodimeric EGFR and HER2 as determined for all three membrane regions are summarized in Figure 7. The plots show that for both receptors, the distributions between active states (dimers) and inactive states (monomers) have similar regional preferences. Generally, the fraction of receptors in the active state followed the relative levels of *ρ*_R_, with LMP areas having the highest dimer shares of 86% for HER2 and 96% for EGFR, FSM areas had approximately equally distributed dimers and monomers, and lamellipodia had the lowest dimer shares at 23% for HER2 and 12% for EGFR. A similar trend, i.e., a trend following local *ρ*_R_, was also found for HER2 in heterodimers, however, these shares were much smaller, ranging from the highest values of 6% in LMP, 3% in FSM, and <1% in lamellipodia. In contrast, a rather constant percentage of ~10% EGFR was found in homodimers in all three regions. This invariant and substantial share of EGFR homodimers was surprising. First, because it was independent of local *ρ*_R_, second, because the cells were grown in media without EGFR, and these detected EGFR homodimers were thus ligand-independent dimers, and third, the presence of an overall > 20-fold surplus of HER2 versus EGFR should theoretically lead to much lower percentages of EGFR homodimers and higher shares of EGFR in heterodimers.

To examine if SKBR3 cells with the highest EGFR *ρ*_R_ (>*P*84) had different distribution profiles of dimeric and monomeric receptors in LMP and FSM, their LMP and FSM data were separately analyzed. In FSM, this subpopulation of cells showed slightly higher percentages of EGFR and HER2 in heterodimers, but in LPM a doubling of the EGFR share in homodimers and of HER2 in heterodimers was found (see Figure S4). These results support the hypothesis that EGFR is a preferred dimerization partner for itself than for HER2 and that in regions with higher EGFR *ρ*_R_ EGFR homodimers become increasingly important, even in an environment with much more HER2 available than EGFR.

## 4. Discussion

A single molecule-based strategy was developed for the subcellular detection and absolute quantification of endogenously expressed EGFR and HER2 *ρ*_R_, their respective ratio, and their dimerization behavior, including a quantification of the heterogeneity of these receptor features in breast cancer cells. The mandatory requirement for absolute quantification was the determination of the labeling efficiency *η*.

We found that the distribution patterns of receptor *ρ*_R_, as well as their dimerization behavior, differed between distinct subcellular regions of the plasma membrane, LMP, FSM, and lamellipodia, each known to exhibit characteristic topographies and dynamics. Generally, EGFR and HER2 were non-homogenously distributed over the plasma membrane and had 3- to 6-fold higher average *ρ*_R_ in LMP, the most dynamic membrane regions of SKBR3 cells, compared to two other types of membrane regions. The latter finding matches earlier results of locally increased levels, and a co-localization of EGFR and HER2 in membrane protrusions of SKBR3 cells [16]. Due to the known values of the labeling efficiency *η* for both receptors, it was possible to calculate the absolute *ρ*_R_ despite differing *η* between the larger and the smaller QD label. The obtained *ρ*_R_ values for both labels reveal the large range by which these vary between cells and even between different regions of a single cell, as well as between both receptors, thereby highlighting the involved phenotypic heterogeneity. The obtained *CV*s (Table 1) quantify the level of heterogeneity. Their average value is consistent with the reported value of 0.4 for the median *CV* of protein expression in cancer cell lines [52], which lies above the range of 0.1–0.3 for protein expression in non-cancer cells [53]. Our analysis also facilitated quantifying the heterogeneity of the local HER2/EGFR *ρ*_R_ ratios, which yielded *CV* values between 0.7 and 1.0. We interpret these *CV* values in the sense that in cancer cells, the interaction of two different protein species can reach a higher level of heterogeneity than the heterogeneity found for each single protein species. In HER2-overexpressing breast cancer patients intra-tumoral heterogeneity of HER2 expression, as measured on a relative level, was found to significantly correlate with disease progression [54,55].

A notable finding of our study was the high *ρ*_R_ of HER2 on LMP, with an average of >1100/μm^2^ and a maximum of almost 3000/μm^2^. EGFR was found with much lower average densities of 70/μm^2^, reaching a maximum value of ~230/μm^2^. The lowest receptor *ρ*_R_ was detected on lamellipodia, with averages of 12/μm^2^ for EGFR and ~250/μm^2^ for HER2. These absolute numbers show that the *ρ*_R_ of both receptors can locally reach similar values, despite the 25-fold higher average *ρ*_R_ of HER2. Based on the absolute values of *ρ*_R_ and HER2/EGFR *ρ*_R_ ratios, we propose the hypothesis that in regions with highest EGFR *ρ*_R_ and lowest HER2/EGFR*ρ*_R_ ratios, the strength of EGFR signaling can reach or might even outperform HER2 signaling. In patients with HER2 overexpressing tumors, the detection of cell subpopulations with a low HER2/EGFR *ρ*_R_ pattern could probably indicate a need to supplement the standard HER2-targeting therapy with an EGFR-targeting drug. Another possible new benefit of knowing the absolute *ρ*_R_ of EGFR and HER2, and their *CV*s, is that this information would permit the calculation of an estimate of the total number of receptors in the patient tumor. Because the efficacy of targeted antibody therapies depends on the expression level of the target molecules that will capture and bind the antibodies on tumor cells [56,57], this information could be used to individually optimize the dose of the applied drug [58].

To determine if the three dynamically distinct membrane regions deviated also with respect to the dimerization behaviors of the two receptors, we calculated the pair correlation function *g*(*r*) of all label positions, yielding the typical peaks indicating the presence of homodimers of EGFR, respect. HER2 [17,47,48,50]. Ligand-independent EGFR homodimers were found in all three regions at percentages of ~10%. The existence of these unliganded or pre-formed EGFR homodimers has been suggested earlier using other techniques [59,60,61,62]. Here we confirm their existence, even in the presence of a 25-fold surplus of another potential dimerization partner. The significant share of EGFR homodimers might be due to a higher binding affinity between two unliganded EGFR than between an unliganded EGFR and HER2 [63]. Our data do not support the published hypothesis of an overexpression-induced mechanism for ligand-independent EGFR homodimerization [62] because EGFR expression in SKBR3 cells is even lower than in normal breast tissue, as is generally the case in human breast cancers [35]. In contrast to EGFR, average percentages of HER2 homodimers were significantly higher, starting with values of ~20% in lamellipodia, and reaching averages as high as 80% in LMP. This result confirms our earlier reports of a high emergence of HER2 homodimers in membrane ruffles [17,47,64]. We can thus conclude that large amounts of this orphan receptor exist in a signaling active state in specialized membrane regions of overexpressing tumors cells, despite a predicted instability and short lifetime of the HER2 homodimer [45].

Besides the two homodimers, we also confirmed the existence of heterodimers through the analysis of the combined dual label position data with an expanded pair correlation function *g*_2_(*r*). EGFR was found to assemble in heterodimers at percentages of >40% in clustered regions, and >80% in LMP, only in lamellipodia was the share on average just 3%. As expected for HER2, the relative shortage of EGFR as a dimerization partner resulted in low average heterodimer percentages, with the highest average of 6% occurring in LMP. These rather low percentages of heterodimers in the total receptor population might be partly compensated for through the higher signaling strength of heterodimers compared to EGFR- and HER2 homodimers [4], likely due to their extended signaling time [63]. In cancer cells, EGFR heterodimers play an important role in proliferation and migration behavior [65,66,67], which is in accordance with findings from patient studies revealing a significantly adverse disease outcome in cancer patients with overexpressed levels of both receptors compared to those with overexpression of only one receptor species [66,67,68,69]. Although the HER2/EGFR ratios in the three types of membrane regions were similar, the EGFR shares in heterodimers showed large, subregional differences, with the highest shares in LMP. It seems that a yet unknown, unique molecular mechanism, which is localized in LMPs and to a lesser extent in FSM, actively promotes the heterodimerization of ligand-independent EGFR with HER2. The shares of dimeric EGFR in LMP indicate that in these dynamic membrane regions, the majority of EGFR is in a fast-starting position for ligand-induced signaling as soon as ligands are available [21,62], or they might already be in a signaling-competent state, as shown in a HER2 and EGFR overexpressing cell model [70]. Future elucidation of the underlying mechanism driving receptor dimerization in LMP should point to new ways of inhibiting EGFR heterodimer formation and thus increase the survival chances of patients with overexpression of both receptors.

The focus of this study was on the dimerization of EGFR and HER2, as both receptors have pivotal clinical importance, but several other possible dimerization partners, from the same HER family, as well as from other receptor kinase subfamilies, can also interact with these receptors [71]. Quantification of such rather “promiscuous” interactions of EGFR and HER2 might be desired in the future, and could be achieved by exchanging EGF or the anti-HER2 affibody with other specific receptor ligands, for instance insulin-like growth factor I [72], or another engineered binding peptide, such as an anti-HER3 affibody [73]. Depending on the availability of specific, small binding molecules, the method could be even extended to measure a broad range of endogenous membrane protein interactions, and it can possibly be combined with a protocol we recently developed for the detection of HER2 in dissociated tumor cells from patient FFPE tissue samples [64]. Quantification of the interaction of receptors in cancer cells from patients might be worth considering in future studies of HER2 and EGFR (over-) expressing patient tumor cells. The results could support future optimized therapeutic concepts for HER2 overexpressing cancer patients with increased EGFR levels, for instance by addition of the EGFR inhibitor gefinitib [66], or of the ErbB receptor tyrosine kinase inhibitor neratinib [74,75] to the current standard HER2 targeting therapy with Trastuzumab and Pertuzumab. However, it should be kept in mind, that EGFR is also involved in oncogenic functions related to cell survival, which are independent of its tyrosine kinase activity [76], and thus likely do not correlate with receptor dimerization.

The strength of the currently presented method is the absolute quantification of receptor dimerization in intact cells, and independent of the underlying receptor *ρ*_R_, which distinguishes it from another protein dimerization detection method, known as proximity ligation assay [77]. The latter light microscopic technique provides a relative read-out from the detection of proteins located within distances up to 40 nm, which is too large to prove direct physical interaction and includes proteins being in close vicinity by random chance. In addition, and similar to other detection methods relying on antibody-binding, steric hindrance leads to signal saturation at higher levels of receptor *ρ*_R_, excluding the use of this assay for quantitative purposes in overexpressing cancer cells [78].

## 5. Conclusions

We demonstrate the ability of our dual-labeling and microcopy method to gather absolute quantitative data on EGFR and HER2 *ρ*_R_, on the respective *ρ*_R_ ratios, and on the percentages of both receptors in dimers and monomers, which can be used to determine their heterogeneity. Mobile regions of the plasma membrane, in particular LMPs, serve as powerful, molecular “partner agencies”, bringing the majority of both receptors into heterodimers and homodimers, whereas, in FSM regions, only half of the receptors assembled into dimers, and in lamellipodia, > 3/4 of both receptor types remained monomeric. The method could be applied in the future as a possible prognostic biomarker detection tool for cancer patients, as well as for finding the optimal personalized dosage of receptor-targeting antibody drugs.

## Figures and Tables

**Table 1:** Characterization of EGFR and HER2 in distinct types of plasma membrane regions of SKBR3 cells. STEM images were analyzed and n QD labels were detected. The determined average values of both receptor *ρ*_R_ are given, and the respective percentages of receptors residing either in EGFR or HER2 homodimers (homo), in heterodimers (hetero), and in monomers (mono). The statistical coefficient of variation (*CV*) of the respective receptor *ρ*_R_ and the ratio between both *ρ*_R_ are also included.

Type	Images	Receptor	*n*	*ρ*_R_	Homo	Hetero	Mono	*CV*	CV of ρ_R_ Ratios (HER2/EGFR)
FSM	102	HER2	78,425	340	0.45	0.03	0.52	0.5	0.8
		EGFR	2636	22	0.11	0.42	0.47	0.8	
LMPs	64	HER2	178,003	1124	0.8	0.6	0.14	0.5	1.0
		EGFR	6065	70	0.12	0.84	0.04	0.7	
Lamellipodia	31	HER2	18,710	255	0.23	0.00	0.77	0.3	0.7
		EGFR	662	13	0.09	0.04	0.88	0.6	

## Data Availability

The datasets used and analyzed during the current study are available from the corresponding author on reasonable request.

## Supplementary Materials

The following are available online at https://www.mdpi.com/article/10.3390/cells10113244/s1, Figure S1: Analysis of possible correlations between EGFR and HER2 *ρ*_R_, between cell surface size and receptor *ρ_R_*, respect. numbers of receptors per cell, Figure S2: Quantitative fluorescence intensity (FI) controls comparing the QD565 FI values of SKBR3 cells after single HER2 labeling and after dual EGFR/HER2 labeling, with both protocols performed with cells from the same passage, Figure S3: Determination of the labeling efficiency *η* for QD655-labeling of membrane-bound EGFR, Figure S4: Analysis of receptor fractions in dimers and monomers, as found in a subpopulation of SKBR3 cells with higher EGFR *ρ*_R_ (>*P*84 of the SKBR3 population), Table S1: Receptor dimerization characteristics in distinct types of plasma membrane regions of SKBR3 cells with higher EGFR *ρ**_R_* than average (EGFR/µm^2^ > *P*84)., Additional Materials and Methods: Determination of the labeling efficiency *ρ*_R_ for membrane-bound HER proteins.
