# Supplementary material for: Quantification of EGFR-HER2 Heterodimers in HER2-Overexpressing Breast Cancer Cells Using Liquid-Phase Electron Microscopy
Source: Cells. 2021 Nov 19;10(11):3244. doi: 10.3390/cells10113244 (PMC8623301; doi:10.3390/cells10113244)
Supplement: Supplementary file 1 [file cells-10-03244-s001.zip › cells-1438684-supplementary.pdf]

Supplementary Information

Quantification of EGFR/HER2 heterodimers in HER2-overexpressig breast cancer cells using liquid phase electron microscopy

Diana B. Peckys, Daniel Gaa, and Niels de Jonge

Contents:

- Figures S1-S4
- Table S1
- Supplementary Results
- Supplementary Methods
- Supplementary References

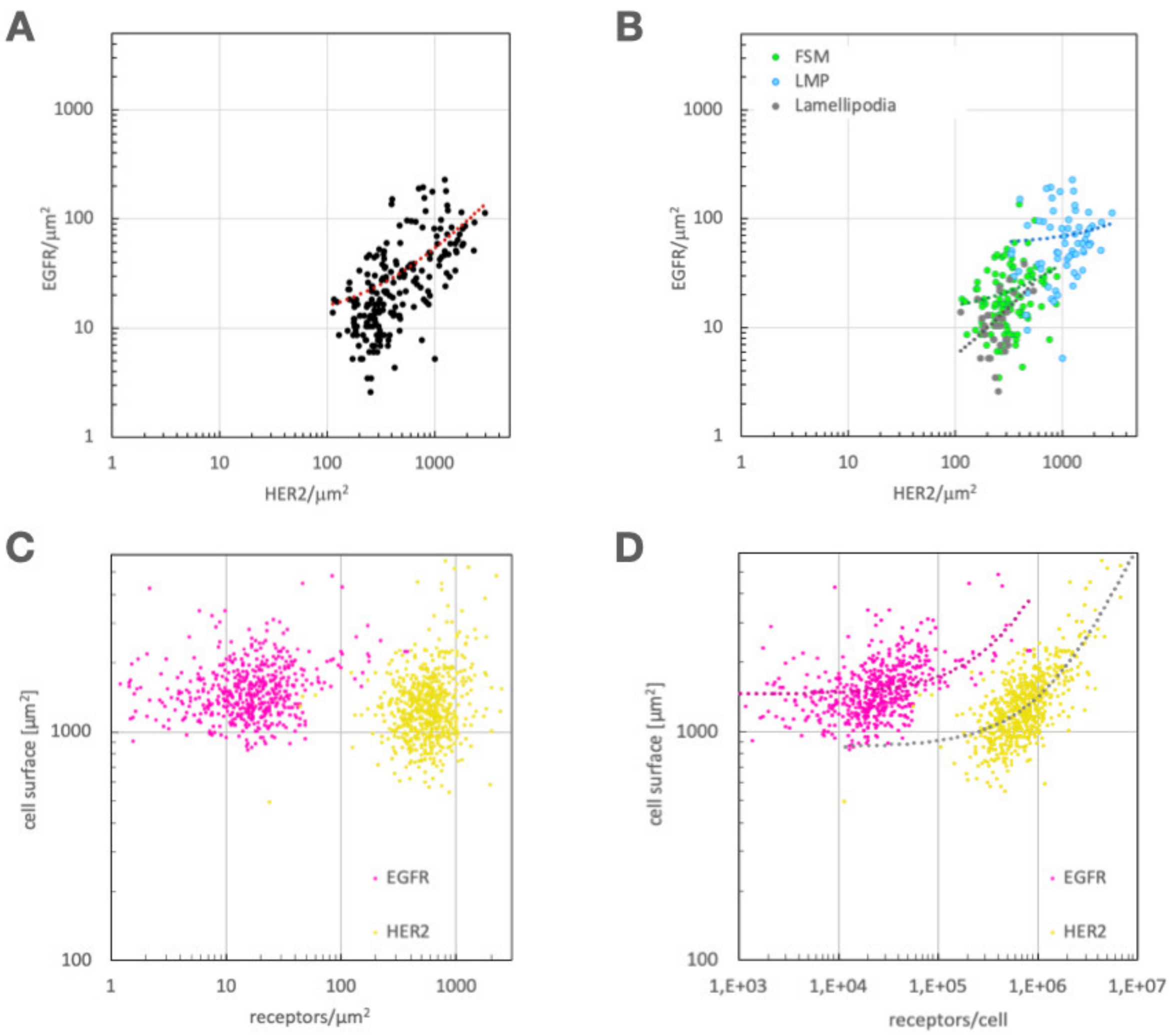

Figure S1. Determination of linear correlations between epidermal growth factor receptor (EGFR) and human epidermal growth factor receptor 2 (HER2) surface density ρ_R, between cell size and receptor ρ_R, and between cell size and total number of receptors per cell. (**A**) Scatter plot of the corresponding EGFR and HER2 ρ_R in all acquired scanning transmission electron microscopy (STEM) images (same data set shown in Figure 4D), linear regression analysis (red dotted line) resulted in Pearson correlation coefficient $R^2 = 0.28$, indicating no correlation. (**B**) The same data as in (**A**) were divided into the three groups of membrane regions and analyzed separately, but none of the regions showed a correlation (lamellipodia: grey dotted line, $R^2 = 0.24$, fine structured membrane (FSM): green dotted line, $R^2 = 0.04$, and large membrane protrusions (LMP): blue dotted line, $R^2 = 0.01$). (C) Scatter plots of single cells labeled with fluorescein (FITC)-conjugated EGF or FITC-conjugated HER2-affibody, showing the determined ρ_R for EGFR and HER2 against the cell's surface area, which were determined from the individual, spherical cells in suspension. Linear regression revealed no correlations (both $R^2 < 0.1$). (**D**) The data set used for the plot in (**C**) was analyzed for a correlation between the total number of membrane-bound receptors per cell and the size of the cell surface, revealing a correlation for HER2 (grey dotted line, $R^2 = 0.64$), but not for EGFR (magenta dotted line, $R^2 = 0.17$).

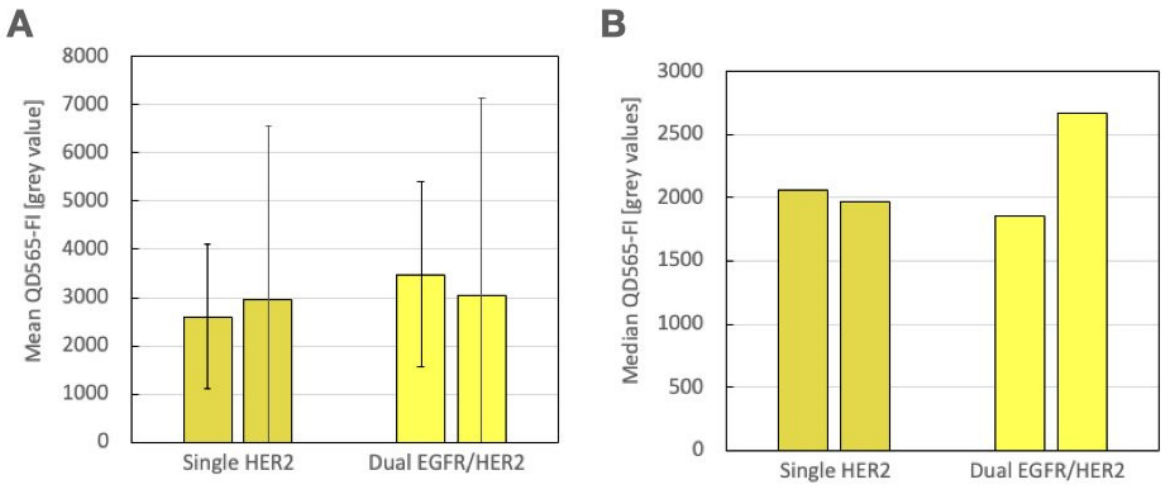

Figure S2. Control experiments testing if the labeling efficiency η, as determined from single labeling experiments of HER2, had changed due to dual labeling of EGFR and HER2. (**A**) The results show that the average HER2 QD565 fluorescence intensity (FI) level, measured in duplicates from single HER2 labeling experiments (dark yellow) was not different from the HER2 QD565 FI signal of duplicates of dual EGFR/HER2 labeling experiments (yellow) (error bars show standard deviations). Each bar represents FI measurements of an area of ~1.2 mm^2 covered with a >60% confluent SKBR3 cell layer containing several hundred cells. The four experiments were done with cells from the same cell passage. (**B**) Also, when computing the median HER2 QD565 FI, similar values were found between the single labeling experiment and the dual labeling experiment. On the basis of these results we concluded that the earlier determined value of the labeling efficiency $\eta = 0.8$ in single HER2 labeling experiments [1] was applicable for the calculation of the HER2 surface density and dimerization in dual labeling experiments.

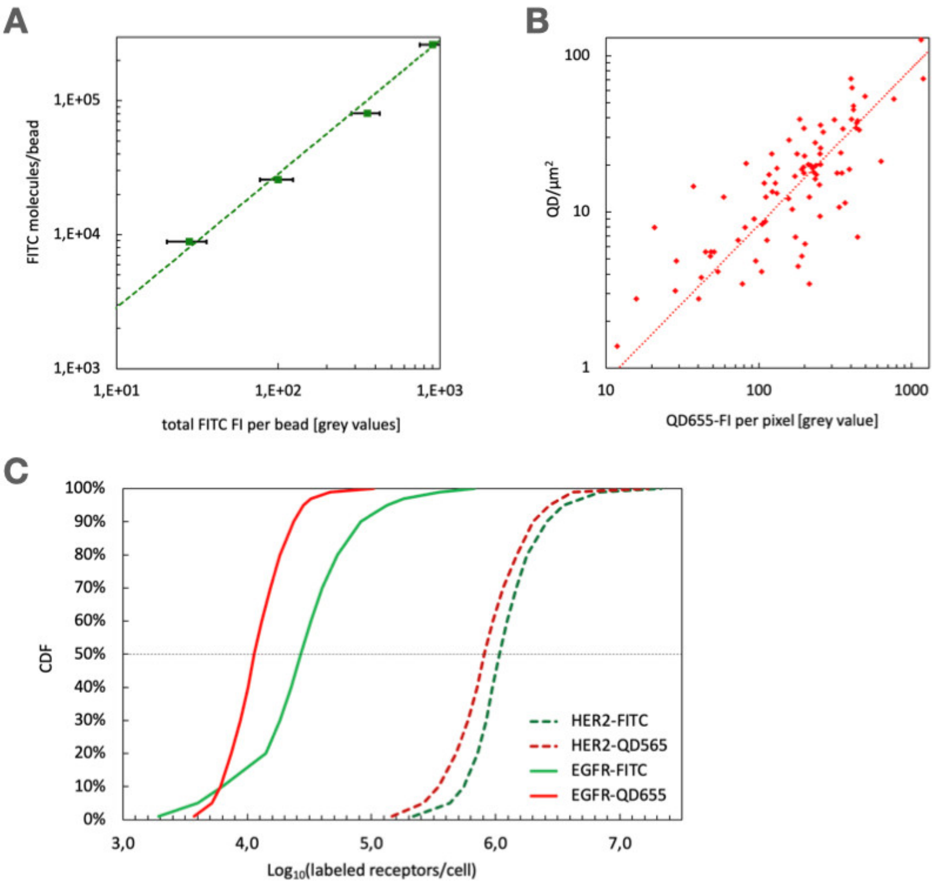

Figure S3. Determination of the labeling efficiency η for QD655-labeling of membrane-bound EGFR using a protocol described elsewhere [1]. (**A**) A FITC-calibration curve was created to prepare the determination of the EGFR/cell distribution in the SKBR3 cell population using the small FITC label, expected to yield $\eta = 1.0$. Standard FITC-beads, usually used for calibration in quantitative flow cytometry, were imaged with fluorescence microscopy using the same settings as for FITC-labeled EGFR on SKBR3 cells. The plot shows the linear correlation of summed FITC-FI values from single FITC beads (n = 65–128 per data point) with defined numbers of FITC molecules, known as molecules of equivalent soluble fluorochrome (MESF). Linear regression ($R^2 = 0.99$) yielded the conversion factor β_{FITC}, required for the conversion of FI values of single cells with FITC-labeled EGFR/cell, into the number of membrane-bound EGFR/cell. (**B**) A similar calibration curve for the conversion of QD655 mean FI/pixel into QD/µm^2, was created from quantitative measurements of correlating fluorescence and STEM images from the same regions of QD-labeled EGFR on SKBR3 cells (n = 87). Linear regression ($R^2 = 0.84$) yielded the conversion factor β_{QD655}, converting mean FI (MFI) values of single cells with QD655-labeled EGFR/cell, into the total number of labeled EGFR/cell, after multiplication with the area size of each cell. (**C**) Cumulative distribution functions *CDF* were calculated for the FITC- (solid, green line, n = 543) and QD655-labeled cells (solid, red line, n = 968). The graphs represent the distribution of membrane-bound EGFR/cell values found in the analyzed single cells of the SKBR3 cell populations. Division of the median value of the QD655-CDF by the one of the FITC-CDF yielded a labeling efficiency $\eta = 0.42$ for QD655-labeling of EGFR. The

dotted, dark green and dark red lines represent the CDF of FITC-, respect. QD565-labeled HER2 on SKBR3 cells, as determined earlier [1]. For this receptor, the smaller label size yielded an almost two-fold higher $\eta = 0.8$.

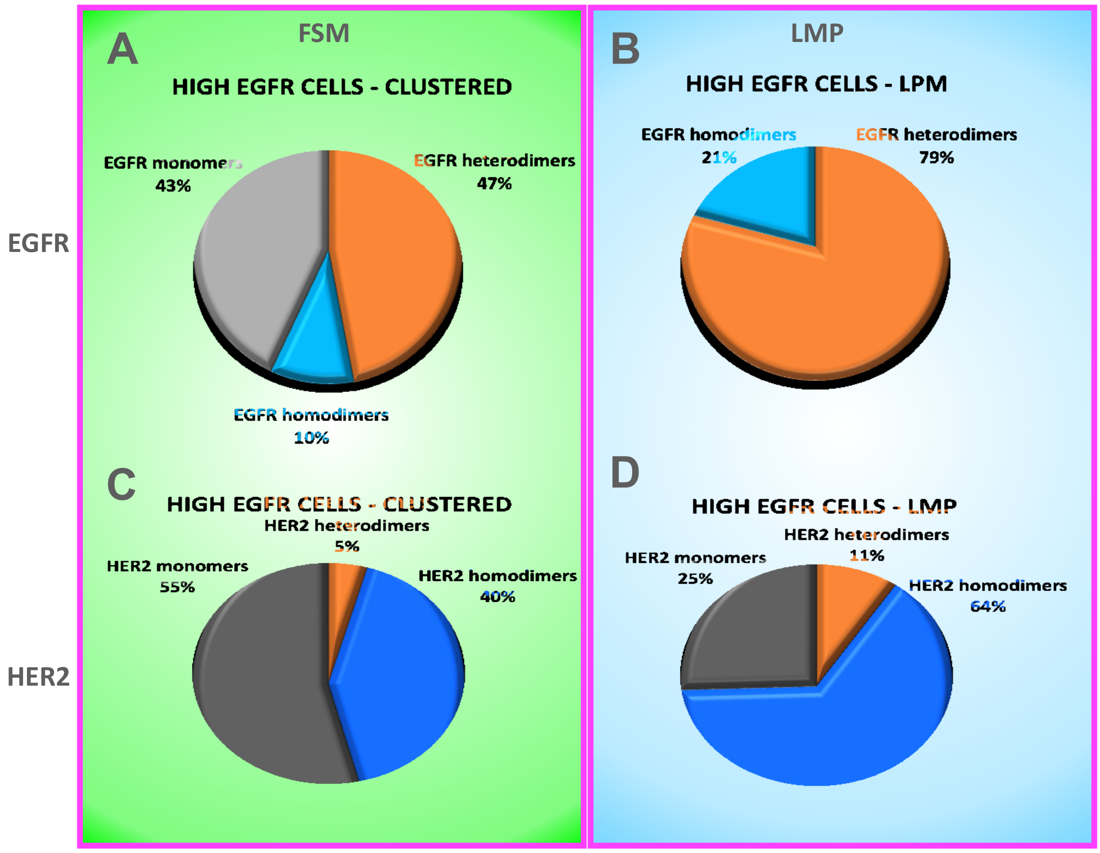

Figure S4. Analysis of receptor fractions in dimers and monomers, as found in a subpopulation of SKBR3 cells with higher EGFR ρ_R (>P84 of the SKBR3 population). (**A**, **C**) Receptor distribution profiles in FSM showing slightly higher fractions of EGFR and HER2 in heterodimers than fractions found the whole SKBR3 population (see Figure 7). (**B**, **D**) In LMP the fractions of EGFR in homodimers and of HER2 in heterodimers were approx. two-fold higher, compared to the respect. receptor fractions in the whole SKBR3 cell population (see Figure 7 of the main manuscript). These results indicate not only that this subpopulation has a higher total number of EGFR, due to the higher EGFR ρ_R, but also that the relative participation of EGFR in all receptor dimers was disproportionately increased.

Table S1. Receptor dimerization characteristics in distinct types of plasma membrane regions of SKBR3 cells with higher EGFR ρ_R than average (EGFR/µm^2 >P84)

Type of Plasma Membrane	**Receptor**	**Number of detected QD labels**	**Receptors/ µm^2**	**Fraction in homodimers**	**Fraction in heterodimers**	**Fraction in monomers**	**Average ρ_R ratios (HER2/ EGFR)**
FSM	HER2	26,418	327	0.4	0.05	0.55	9.3
	EGFR	1,43	35	0.1	0.47	0.43	
LMP	HER2	53,432	849	0.64	0.11	0.25	7.1
	EGFR	3,490	120	0.21	0.79	0	

Supplementary Results

Determination of the labeling efficiency η for membrane-bound HER proteins

To determine the absolute values of the surface density of EGFR-HER2 heterodimers, as well as the fraction of EGFR and HER2 assembling in heterodimers, homodimers, or remaining as monomers, knowledge of the labeling efficiency η is required for each of the two receptors. Using the same cell line we recently developed a method for determining η for QD labeled HER2 [1], from single labeling experiments of HER2 only. Comparing the signal intensity of QD565-labeled HER2 on SKBR3 cells after single labeling experiments versus after dual labeling experiments showed similar values for the fluorescence intensity (FI) (see Figure S2). We thus concluded that η for the applied QD-labeling of HER2 after prior QD-labeling of EGFR remains the same as the previously determined η of 0.8.

To determine η for the EGFR QD-labeling we followed the same principle as done for HER2 [1], except that the biotin-conjugated HER2-specific affibody was replaced by biotin-conjugated EGF. Our approach resembles quantitative flow cytometry and relies on log normal distributions of proteins in cells [2]. We first measured the cell population distribution of FITC-labeled EGFR per cell (HER2-FITC/cell), serving as standard for η = 1.0, or 100%. For the conversion of measured FITC FI into the number of FITC molecules a calibration curve was created from analysis of LM images of FITC calibration beads, recorded with the same imaging settings as used for the FITC-EGF labeled cells (see Figure S3A). The calculated slope value β_{FITC} of the linear regression converts the summed FITC FI values per bead into the amount of FITC molecules per bead. Next, a second calibration curve was created to convert the QD655 FI of QD-labeled EGFR on SKBR3 cells into the underlying surface density of QD bound to EGFR at the plasma membrane. This curve (shown in Figure S3B) was created by quantitative and correlative LM and STEM, measuring EGFR-QD655 ρ_R and FI per pixel from the same, dual labeled SKBR3 cells on microchips with electron-transparent SiN membrane windows. Quantitative LM was used to gather QD FI values of the QD655 channel from regions of interest (ROI) of selected cells. The ROI were selected at the locations of previously recorded STEM images from the same cells, each ROI included an area of a few µm^2. The

measured QD655 FI values were plotted against the surface density of QD655 in the corresponding STEM images, which were automatically determined by our STEM image processing software. The calculated linear regression yielded the slope value β_{QD655}.

For calculating the cumulative distribution function (CDF) for FITC-EGFR on SKBR3 cells, the cells were labeled in cell suspension, live, and on ice, immediately followed by imaging with quantitative LM. Randomly chosen single cells were manually encircled to define the ROI of each cell, from which the surface area and FI values were measured. The cell suspension was chosen because it simplified analysis of the spherical cells, especially of those exhibiting a relatively dim FI. β_{FITC} was used as conversion factor to determine FITC-EGFR/cell, and a CDF was calculated from >500 cells (solid, green line in Figure S3C). Note that the slight deviation of the FITC curve for cells <*P20* (corresponding to <14,000 FITC-EGFR/cell), is likely due to the low level of the FITC FI being close to the FI background values.

For calculating the CDF for QD-labeled EGFR per cell (QD655-EGFR/cell) a series of fluorescence images from adherently grown cells and dual labeled cells in dishes was recorded and automatically analyzed with a segmentation tool and FI quantification program, yielding the surface area, and the FI values for ~1,000 cells. These values were then multiplied and converted with β_{QD655} to calculate EGFR-QD/cell values, which were then used to calculate the CDF (solid, red line in Figure S2C). The median of the CDF for QD655-EGFR/cell was 1.1×10^4, the median of the FITC-EGFR/cell was 2.7×10^4 EGFR/cell, resulting in η =0.4 for QD-labeled EGFR, a similar value as found for QD655 labeling of HER2 [1]. For comparison, the earlier determined CDFs for HER2 labeled with FITC (dotted, green line in Figure S2C) and with QD565 labeling (dotted, red line in Figure S2C) are also displayed. These HER2 labeling protocols yielded median values of 1.1×10^6 for FITC-HER2/cell, respect. 8.1×10^5 for QD565-HER/cell with η ~0.8, twice as high as for QD655-EGFR, probably due to a weaker steric hindrance of the smaller QD565.

Supplementary Methods

Segmentation of cells in fluorescence images of labeled EGFR.

The individual cells in the fluorescence image were segmented to determine their area and intensity as needed for the calculation of η of EGFR. In a preprocessing step (performed in ImageJ), the quality of the fluorescence images was improved by increasing the contrast, performing an edge-enhancing smoothing with the Anisotropic Diffusion 2D tool of ImageJ, and setting the background pixel values to zero. The distinction between signal- and background was based on a user-defined threshold, which was determined easily using the histogram information. To segment the fluorescence images of the cells, the program CellProfiler was used [3]. Firstly, the approximate middle positions of the cells were annotated manually as point annotations, and a binary image with small dots at those positions was created. These dots were used as primary objects, based on which the surrounding cells in the preprocessed fluorescence image were segmented by thresholding with an adaptive minimum cross-entropy setting. To be able to subtract the background

intensity in the measurements, several reference areas were defined in the background area. The background-corrected mean fluorescence of each cell and the cell's areas were measured for all each cell in the original fluorescence image using the cell's outline information obtained from the cell profiler.

Supplementary References

1. Peckys, D. B.; Quint, C.; Jonge, N. Determining the Efficiency of Single Molecule Quantum Dot Labeling of HER2 in Breast Cancer Cells. *Nano Lett.* **2020,** 20, 7948–7955.
2. Heins, A.-L.; Johanson, T.; Han, S.; Lundin, L.; Carlquist, M.; Gernaey, K. V.; Sørensen, S. J.; Eliasson Lantz, A. Quantitative flow cytometry to understand population heterogeneity in response to changes in substrate availability in Escherichia coli and Saccharomyces cerevisiae chemostats. *Front. Bioeng. Biotechnol.* **2019,** 7, 187.
3. McQuin, C.; Goodman, A.; Chernyshev, V.; Kamentsky, L.; Cimini, B. A.; Karhohs, K. W.; Doan, M.; Ding, L.; Rafelski, S. M.; Thirstrup, D.; Wiegraebe, W.; Singh, S.; Becker, T.; Caicedo, J. C.; Carpenter, A. E. CellProfiler 3.0: Next-generation image processing for biology. *PLoS Biol.* **2018,** 16, e2005970.
